# Translating Telehealth Communication Research Into Patient-Centered, Implementable Practice

**DOI:** 10.2196/93690

**Published:** 2026-03-19

**Authors:** Rachel Pittmann, Paula D Koppel, David Barrett

**Affiliations:** 1 Center for Interprofessional Education and Practice, MGH Institute of Health Professions, 36 1st Avenue, Charlestown, MA, 02129, United States, 1 (617) 724-9852; 2 School of Nursing, Duke University, Durham, NC, United States; 3 Department of Health Sciences, University of York, Heslington, York, United Kingdom

**Keywords:** telemedicine, telehealth, patient-centered communication, videoconferencing, phone-based virtual care

## Abstract

Understanding both patient and clinician perspectives on communication challenges in virtual primary care consultations is important to ensure safe and effective care. This commentary reviews the important work of Alboksmaty and colleagues, highlighting their contributions and noting some limitations. To further support translation of clinical practice–based recommendations from this original research, this commentary relates the study findings to patient-centered communication frameworks and evidence-based virtual communication strategies. This commentary also extends the helpful mitigation strategies offered in the original research, demonstrating how they apply to the Expert Recommendations for Implementing Change (ERIC) implementation framework to facilitate organization-based change. The need for additional research differentiating phone-based and videoconferencing-based primary care consultation communication strategies is highlighted.

## Introduction

We congratulate Alboksmaty and colleagues [1] on their interesting study analyzing the communication challenges that occur during virtual consultations and thank them for generating mitigation strategies to address these challenges. Using 2 frameworks, the Shannon Weaver communication model (SWCM) and the capability, opportunity, motivation, and behavior (COM-B) model, to support their analyses, the researchers analyzed the data they collected during focus groups with UK-based general practitioners and patients to understand the communication barriers and gaps in virtual consultations. The SWCM and COM-B provided an interesting perspective when applied to telehealth communication. After reviewing this study closely, we offer some guidance for viewing and implementing the findings in practice.

## Disaggregate Phone-Based and Videoconferencing-Based Virtual Consultation Results

In Alboksmaty and colleagues’ study [1], phone-based and videoconferencing-based consultations were considered together under the umbrella heading of “virtual” consultations. Our view would be that treating virtual consultations as a homogeneous entity and combining these two modalities into one analysis limits the ability to fully interpret the communication challenges. For example, the first theme in the findings highlights the patients’ and clinicians’ challenges in interpreting emotional and clinical cues, resulting in feelings of disconnection related primarily to phone-based virtual care. Similarly, the second theme of compromised clarity when discussing complex or sensitive issues was also more relevant to phone-based virtual care, as was the third theme, relating to challenges in trust and confidence centered around the absence of nonverbal interactions. We propose that separately analyzing phone-based and videoconferencing-based virtual care could provide a clearer understanding of the unique strengths and challenges of each modality. Such an approach might have led to different interpretations of the fifth and sixth themes—aligning virtual modalities with clinical needs and balancing contextual expectations with patient preferences. Disaggregating the data into phone-based and videoconferencing-based modalities could better support shared decision-making between clinicians and patients by helping to determine which option is most appropriate for a given clinical scenario. This approach aligns with recent calls for researchers to directly compare the use of phones and videoconferencing in primary care to inform a structured process for modality selection [2].

## Use Patient-Centered Communication Models and Principles in Telehealth Research

We would have found it helpful to understand the rationale for the researchers’ selection of the SWCM to support their analysis. The SWCM was originally a model developed in the mathematical theory of communications and, though it has been used in health care research and education, we do not believe it enables a holistic analysis of the complex nature of human interactions between clinicians and patients. Using the Roter interaction analysis system, often leveraged to measure verbal and nonverbal communication patterns in health care, including telehealth [3], or the patient-centered communication (PCC) framework, associated with quality in health care [4], may have supported more meaningful interpretation of the data.

We offer an expansion of Alboksmaty et al’s [1] findings by aligning them with PCC principles whereby clinicians empathically engage with patients’ needs and values and share in decision-making. PCC, as described by Moser et al [5], is organized around 7 core functions, which include ensuring adequate time and attention for patients to ask questions and address their emotional needs (functions 1, 2, and 6), sharing decision-making (function 3), enabling patients to understand how to take care of their health (function 4), providing clinical explanations that are understandable (function 5), and supporting patients in managing feelings of uncertainty (function 7). PCC has been shown to enhance patient trust and medication adherence as well as overall patient satisfaction [6].

In Table 1, we align Alboksmaty et al’s [1] study findings with the PCC core functions and evidence-based, patient-centered virtual communication tools. As can be seen in the table, training and skill development for virtual consultations relate to 4 of the 6 study themes, reflecting the importance of building capacity (ie, knowledge and skill) for clinicians, patients, and organizations. Clinicians should consider that phone-based and videoconferencing-based consultations require distinct communication adaptations, and that specific needs and preferences of patients may also require accommodation. We encourage clinicians to consider their role in the development of the patient-clinician relationship and reference evidence-based telehealth PCC strategies [7-9] in the table to ensure the virtual environment supports patients’ preferences and needs.

**Table 1.** Study findings in relation to patient-centered communication (PCC) challenges, mitigation strategies, and evidence-based virtual communication tools.

Communication challenges and associated mitigation strategies identified by Alboksmaty et al [1]	PCC functions at risk if mitigation strategies are not used^a^	Evidence-based, patient-centered virtual communication tools [7-9]
Theme 1 challenges: missed cues and misinterpretations (sender-encoder) Theme 1 strategies: training and skill development for virtual consultations (capability)	Question (function 1) Decisions (function 3) Understood (function 4) Explain (function 5)	Create a safe, private, and comfortable environment for the visit in which the patient and clinician can adequately see and hear one another. Maintain attentiveness and presence. Personalize communication based on patient’s information needs (literacy, preferred learning style, desired level of detail, sensory challenges). Use summarizing statements and open-ended questions. Avoid medical jargon. Check for understanding (teach-back and follow-up questions). Ensure adequate time for discussion and questions. Demonstrate empathy using verbal and, whenever possible, nonverbal means.
Theme 2 challenge: compromises related to lack of clarity in virtual communication (message) Theme 2 strategy: addressing barriers to effective communication (opportunity)	Question (function 1) Decisions (function 3) Understood (function 4) Explain (function 5)	Maintain attentiveness and presence. Personalize communication based on patient’s information needs (literacy, preferred learning style, desired level of detail, sensory challenges). Use summarizing statements and open-ended questions. Avoid medical jargon. Check for understanding (teach-back and follow-up questions). Ensure adequate time for discussion and questions. Demonstrate and clearly state what is being asked of patients during assessment.
Theme 3 challenges: technical issues, poor connectivity, and accessibility issues (channel) Theme 3 strategies: training and skill development for virtual consultations (capability); triage and risk mitigation (opportunity); addressing barriers to effective communication (opportunity); training and resources to support patient preparedness (opportunity and motivation)	Understood (function 4) Time (function 6)	Have a working knowledge of telehealth technologies and problem-solving skills. Demonstrate a willingness to adapt to changing situations with ease and inform patients of a backup plan for technology failures. Demonstrate respect for the patient challenges that are associated with virtual care. Ensure patients know how to proceed when technology issues arise.
Theme 4 challenge: lack of trust in discussing complex needs (receiver-decoder feedback) Theme 4 strategies: patient and clinician preferences in consultation modalities (motivation); training and skill development for virtual consultations (capability)	Question (function 1) Attention (function 2) Decision (function 3) Understood (function 4) Explain (function 5) Time (function 6) Feelings (function 7)	Develop a joint agenda and an agreement on roles and expectations. Maintain attentiveness and presence. Take time to ensure a safe and comfortable environment (ensure privacy, create an interpersonal connection with the patient). Demonstrate empathy using verbal and, whenever possible, nonverbal means.
Theme 5 challenge: feeling less empathy and emotional engagement from clinician (receiver-decoder feedback) Theme 5 strategies: training and skill development for virtual consultations (capability)	Attention (function 2) Understood (function 4) Time (function 6) Feelings (function 7)	Maintain attentiveness and presence. Intentionally create an interpersonal connection at the beginning of each visit. Ensure the technology and the virtual space facilitate interpersonal connection (ie, eye contact, minimal distractions, adequate lighting). Magnify use of active listening techniques (verbal and nonverbal). Heighten self-awareness to ensure behaviors reflect empathy and care. Demonstrate empathy using verbal and, whenever possible, nonverbal means.
Theme 6 challenge: social norms, cultural factors, and age (context)	Attention (function 2) Decision (function 3) Understood (function 4) Explain (function 5) Time (function 6) Feelings (function 7)	Demonstrate respect for the patient challenges associated with virtual care. Demonstrate willingness to adapt to changing situations with ease and assist patients with technology. Personalize communication approach as noted above to accommodate patient needs and/or preferences.
Theme 7 challenges: lack of patient autonomy and inclusivity leading to decreased engagement and willingness to participate in virtual care	Question (function 1) Attention (function 2) Decision (function 3) Understood (function 4) Explain (function 5) Time (function 6) Feelings (function 7)	Demonstrate respect for the patient challenges associated with virtual care. Demonstrate a willingness to adapt to changing situations with ease and assist patients with technology. Personalize communication approach as noted above to accommodate patient needs and/or preferences.

a Full description of the PCC core functions [5]: (1) question—adequate time for patient to ask all health-related questions; (2) attention—adequate attention to patients’ feelings and emotions; (3) decision—shared decision-making; (4) understood—ensuring patient understands how to manage their health; (5) explain—ensuring patient understands what clinician explains; (6) time— ensuring there is adequate time spent with the patient; (7) feelings—adequate time to manage patients’ feelings of uncertainty about their health.

## Use Implementation Frameworks to Facilitate Transfer of Research Findings to Clinical Practice

To further facilitate the translation of Alboksmaty et al’s [1] findings and the associated evidence-based, patient-centered virtual communication tools into clinical practice, we propose using strategies from the Expert Recommendations for Implementing Change (ERIC) implementation framework [10]. Given the challenges, mitigation strategies, and evidence-based tools shown above, we propose using several ERIC strategies at both the individual clinician level and the organizational level for implementation of mitigation strategies. Specifically, creating a community of telehealth practitioners composed of champion clinicians, who will develop educational materials and outreach initiatives and who will model and simulate change prior to the implementation phase, supports the effective implementation of strategies into practice. Additionally, inviting patients as partners in the community of practice enables their voices to be central to the patient-centered change implementation.

Alboksmaty et al [1] analyzed telehealth communication challenges identified by clinicians and patients using unique frameworks. This commentary found it was feasible to apply their findings to the PCC and ERIC models, providing additional support to students and clinicians adapting their communication to telehealth. Future research focused on better differentiating communication strategies for phone-based and videoconferencing-based consultations is necessary to maximize the quality of virtual care.

## Abbreviations

COM-B: capability, opportunity, motivation, and behavior
ERIC: Expert Recommendations for Implementing Change
PCC: patient-centered communication
SWCM: Shannon Weaver communication model
